# Assessment of the Retinal Vessels in Keratoconus: An OCT Angiography Study

**DOI:** 10.3390/jcm11112960

**Published:** 2022-05-24

**Authors:** Adam Wylęgała, Dominika Szkodny, Rafał Fiolka, Edward Wylęgała

**Affiliations:** 1 Health Promotion and Obesity Management Unit, Department of Pathophysiology, Faculty of Medical Sciences in Katowice, Medical University of Silesia, 40-635 Katowice, Poland; 2 Ophthalmology Department, Railway Hospital, 40-760 Katowice, Poland; dominikacholewa1@gmail.com (D.S.); fiolkarafal@gmail.com (R.F.); ewylegala@sum.edu.pl (E.W.); 3 School of Medicine with the Division of Dentistry, Zabrze Medical University of Silesia, 40-055 Katowice, Poland

**Keywords:** FAZ, vascular density, CCT, OCT-A

## Abstract

This study investigated vascular density and foveal avascular zone (FAZ) parameters using optical coherence tomography angiography (OCT-A) in patients with keratoconus (KC). Participants with KC and healthy controls were included and underwent best-corrected visual acuity (BCVA), keratometry, anterior segment OCT, and macular OCT-A examinations. Of the 70 subjects (mean age 42.9 ± 15.31 years), 79 KC and 47 healthy eyes were included. Significant reductions in the KC group were recorded for the FAZ area, with a mean (±SD) of 0.19 ± 0.12 vs. 0.25 ± 0.09 mm^2^ *p* < 0.001. Central vascular density in KC patients was lower compared with the controls: 6.78 ± 4.74 vs. 8.44 ± 3.33 mm^−1^ *p* = 0.049; the inner density was also decreased in the study group (13.64 ± 5.13 vs. 16.54 ± 2.89 mm^−1^, *p* = 0.002), along with the outer density (14.71 ± 4.12 vs. 16.88 ± 2.42 mm^−1^, *p* = 0.004) and full density (14.25 ± 4.30 vs. 16.57 ± 2.48) *p* = 0.003. Furthermore, BCVA was positively correlated with central vascular density (R = 0.42 *p* = 0.004, total R = 0.40, *p* = 0.006) and inner density (R = 0.44, *p* = 0.002) in patients with KC but not in controls. Additionally, we found a correlation between K2 and inner vascular density (R = −0.30, *p* = 0.043) and central epithelium thickness and outer density (R = 0.03, *p* = 0.046). KC patients had lower macular vascular density and a smaller FAZ than healthy participants. The BCVA in KC patients was correlated with the vascular density.

## 1. Introduction

Keratoconus (KC) is a progressive, ectatic cornea disease with a multifactorial etiology. The essence of this condition is a thinning and protrusion of the cornea, which leads to irregular astigmatism and, often, significant visual impairment. Treatment options for this disease include correction with rigid gas-permeable contact lenses; intracorneal stromal rings; collagen cross-linking (CXL), which slows the progression of the disease; and corneal transplant [1,2]. Symptoms typically occur in adolescence, with corneal thinning and steepening progression until the fourth decade of life. The pathophysiology and etiology of this condition are not fully understood. Researchers have indicated the contribution of genetic and environmental factors, with different biochemical processes and histopathological changes involved. A reduction in the number of stromal keratocytes and collagen fibrils, often with concomitant ruptures in Bowman’s layer and central epithelial thinning with irregular cells, is observed in KC [3]. A decrease in collagen fibril diameter and an increase in proteoglycans have been described in the disease [4]. The condition is classified as non-inflammatory; however, recent studies have demonstrated the presence of proinflammatory cytokines and collagenase in the tear film of patients [5,6]. Since collagen is the major protein of the cornea, the assumption that collagenase enzymes are involved is justified. Although most KC cases are sporadic, numerous genes have been assessed regarding their role in pathogenesis, including the collagen gene [7,8,9]. Associations between KC and many other syndromes and disorders have been described. Additionally, many connective tissue diseases correlate with KC, such as osteogenesis imperfecta, Ehlers–Danlos syndrome, Marfan syndrome, mitral valve prolapse, Mediterranean fever, and joint hypermobility disease, as reported in other studies [3,10]. Alterations in choroidal, stromal, and vascular areas in patients with KC have also been demonstrated [11,12]. KC patients have thicker choroids than healthy controls, which could be associated with the inflammatory mechanism of the disorder [12,13]. Nevertheless, the meaning and cause of these changes remain unclear. Abnormalities and the distribution of collagen lamellae, an essential component of vessels, have been demonstrated in KC. OCT angiography (OCT-A) is a non-invasive imaging tool that allows detailed visualization of the retinal microvasculature to be obtained. Therefore, in this study, we compared the retinal vascularization and corneal parameters of patients with diagnosed KC with those of a control group using SS-OCT [14,15,16].

## 2. Materials and Methods

This observational study included an analysis of 126 eyes performed in the university clinic. This study included 31 women and 39 men. The patients were recruited from the cornea and refractive surgery ambulatory center of the Railway Hospital, Katowice, Poland, presenting for ocular examination. Out of 79 eyes with KC, 45 were previously treated with collagen cross-linking (CXL) at least 14 months before inclusion in the study, and two had intracorneal rings implanted. The mean ± SD preoperative BCVA was 0.23 ± 0.56 logMAR, and the BCVA was 0.17 ± 0.55 logMAR 12 months after the procedure. All the imaging procedures were performed between January 2019 and June 2020. Our study followed the principles of the Declaration of Helsinki, with ethics approval obtained from the Medical University of Silesia Institutional Review Board. The study included 42 KC patients and 28 healthy controls. The current best-corrected visual acuity (BCVA) was measured on the basis of the manifest refraction during the visit.

All patients had a slit-lamp examination and indirect ophthalmoscopy. Patients with no anterior segment disorders other than KC and no posterior segment diseases, such as glaucoma, posterior staphyloma, choroidal neovascularization (CNV), diabetic retinopathy, vascular occlusions, and other retinal dysfunctions, were included. Keratometry and refraction were calculated on a Keratometer KR-1W (Topcon, Tokyo, Japan) Cirrus 5000 (Carl Zeiss Meditec, Dublin, CA, USA), and Angioplex software v 11 was utilized to create OCT angiograms and anterior segment maps with an add-on lens. Central corneal thickness (CCT) and epithelial thickness were calculated using a pachymetry scan. K1 and K2 were calculated using Tomey KR-1W. Each patient had a 6 × 6 mm OCT angiography scan. The Zeiss Angioplex software automatically calculated all OCT-A parameters. Only scans with a quality of more than 7/10 were included in the study. The following parameters were calculated: vascular density and the total vessel length divided by the specific area on a standard 6.0 mm ETDRS map (central 1 mm, inner 3 mm without the center 1 mm, outer 6 mm without the inner and center, and total area of the ETDRS circle). FAZ was described by the size of the three parameters in mm^2^ diameter, that is, a straight line connecting two furthest points and running through the center of FAZ described in mm, and circularity (roundness), which is a value representing how close a shape is to a perfect circle, where 1 represents an ideal circle and 0 represents a straight line [17]. Angioplex displays the vascular density as mm/mm^2^; for conciseness, we used the mm^−1^ notation.

Standard spectral domain OCT scans, including optic nerve 200 × 200 scans and 512 × 512 macular cube scans, were acquired. Posterior scans were obtained after instilling one drop of Tropicamide (Polpharma, Warsaw, Poland). Patients wearing contact lenses were told to abstain from wearing them for 72 h before the examination. Only Polish Caucasians without systemic diseases were included in the analysis to minimize confounding factors. Furthermore, the age and gender did not differ significantly. All measurements were carried out between 14:00 and 17:00.

### Statistical Analysis

Categorical variables were analyzed using chi-square tests. The normality of the data was measured using the Shapiro–Wilk test. Continuous variables were analyzed using analysis of variances (ANOVA). The association between continuous variables was investigated with Pearson’s correlation. We nested the ANOVA for this factor to eliminate bilaterality as a confounding variable. The correlation between the quality index and OCT-A parameters was also tested. Multiple regression was performed to test whether the CXL affected the vascular parameters. Values of <0.05 were considered significant. Statistical analyses were performed using Statistica 13.3 (Tibco, Palo Alto, CA, USA).

## 3. Results

Of the 138 OCT-A scans taken, 8% (8 in KC patients and 4 in controls) were considered below the required quality. The mean age of KC patients was 43.64 ± 15.30 vs. 40.10 ± 14.28 years (Table 1).

**Figure 1:**
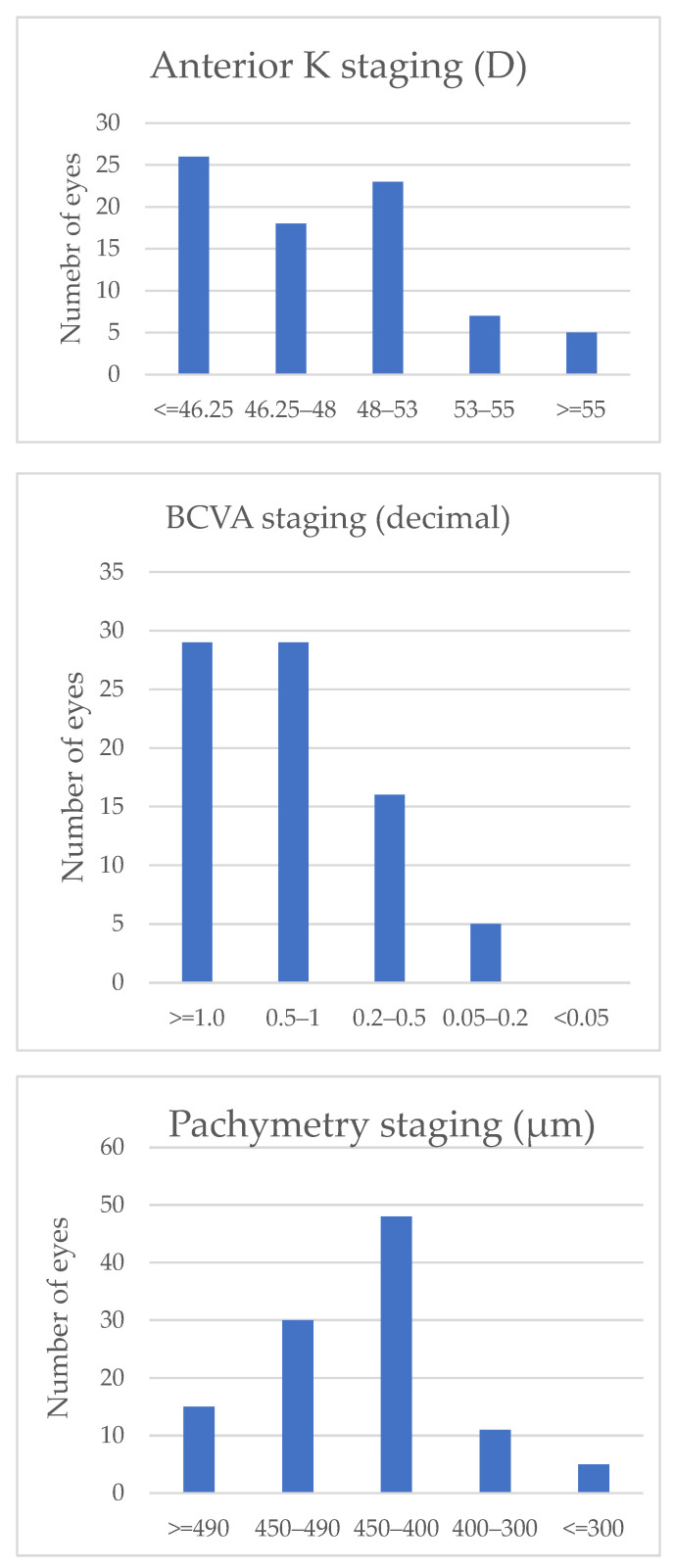
Classification of the eyes included in the study group according to ABCD classification.

Most KC eyes were stage 0 or I, 11 eyes were stage III, and only 10 eyes had parameters classified as stage IV (Figure 1). BCVAs were significantly different between the KC group and the control group and were 0.97± 0.08 and 0.62 ± 0.29, respectively (*p* < 0.001, logMAR: 0.02 ± 0.92 vs. 0.20 ± 0.52).

The mean spherical power in the KC group was −0.89 ± 4.08 D, while the control refractive error was −1.75 ± 2.02 D; the differences were insignificant, contrary to cylindrical power, with a mean of −3.30 ± 2.46 D in KC subjects and −0.92 ± 0.83 D in the normal population (*p* = 0.001).

The average K1 was 44.41 ± 3.92 D in the KC group and 43.22 ± 0.75 in the control group, and the differences were marginally non-significant (*p* = 0.057). However, there was a significant difference between the mean CCTs, which was 478.58 ± 45.27 µm in KC patients and 538.66 ± 29.14 µm (*p* < 0.001) in unaffected individuals.

Other significant changes were observed in the rim area (1.49 ± 0.36 vs. 1.29 ± 0.20, *p* < 0.001), disc area (2.02 ± 0.39 vs. 1.71 ± 0.36, *p* < 0.001), and central corneal epithelium (44.52 ± 7.02 µm vs. 48.83 ± 7.34 µm, *p* = 0.002) in patients with KC vs. controls, respectively. In addition, differences between the minimum and maximum epithelium thickness were almost double for KC (−10.22 ± 6.22 µm vs. −5.17 ± 2.71 µm, *p* = <0.001).

All OCT-A parameters were significantly different between the groups. The difference in central density was marginally significant, with a *p*-value of 0.049 (6.78 ± 4.74 mm^−1^ vs. 8.44 ± 4.33 mm^−1^). The values of KC measurements compared with control measurements for inner, outer, and full density were much lower: 13.64 ± 5.13 mm^−1^ vs. 16.54 ± 2.89 mm^−1^, *p* = 0.002; 14.71 ± 4.12 mm^−1^ vs. 16.88 ± 2.42 mm^−1^, *p* = 0.004; and 14.25 ± 4.30 mm^−1^ vs. 16.57 ± 2.48 mm^−1^, *p* = 0.003, respectively (Table 2).

Overall, in KC group, we observed a negative correlation between age and BCVA (R = −0.46, *p* = 0.001), and RNFL symmetry (R = −0.49, *p* = 0.001), while a positive correlation was observed between K1 and age (R = 0.33, *p* = 0.026). In addition, we found strong positive correlations between the densities measured on the ETDRS circle (range of R = 0.91–0.99). RNFL thickness was significantly correlated with vascular densities in the KC group (range of R = 0.39–0.49) (Table 3).

Further significant correlations were observed between cylinder power and inner, outer, and total vascular density (range of R = 0.40–0.48). BCVA was positively correlated with densities in KC patients but not in healthy controls (Table 4); correlations ranged from r = 0.37 to 0.44 (Table 3). There was no significant correlation between the age, sphere, K1, RNFL symmetry, epithelium max–min, rim and disc area, c/d average, or OCT-A parameters in the KC group. In addition, in controls, neither age, K1, K2, epithelium cube volume, disc area rim area, sphere, cylinder, axis, nor RNFL symmetry was correlated with OCT-A measurements.

Generally, the correlation between the OCT-A and corneal parameters in KC patients was weak or non-significant. The only correlation found was between K2 and inner vascular density (R = −0.30, *p* = 0.043) and central epithelium thickness and outer density (R = 0.03, *p* = 0.046). There was a very high correlation between RNFL symmetry and the inner, outer, and total vascular density in controls (range of R = 0.92–0.97).

Furthermore, the quality index showed no significant correlation between vascular parameters in the KC or the control group.

### Multiple Regression

No significant association was found between the FAZ area, diameter, and circularity; central, inner, and outer density; or a previous CXL procedure (*p* = 0.44). Furthermore, the analysis revealed that KC staging was not associated with the vascular density parameters (*p* = 0.17).

## 4. Discussion

We showed that OCT-A parameters were significantly different between patients with KC and healthy controls. Another interesting finding was that BCVA was positively associated with vascular density, suggesting a possible link between the architecture of the vessels and visual function. As the BCVA in the KC group was significantly lower because of significant astigmatism, decreased vascular density might have impaired retinal function.

KC is a chronic ectatic disease of the cornea. The onset of KC usually begins in the second decade of life. Many hypotheses regarding the origin of this disorder have been proposed, including an inflammatory origin [6]. In addition, KC has been associated with basement membrane distortion [5], sleep apnea, Down syndrome, and allergic diseases, such as asthma, allergic conjunctivitis, and allergic rhinitis [18]. However, a large study showed no association between sleep apnea and KC [19].

Some alterations in posterior segment abnormalities in patients with KC have also been reported. For example, the subfoveal choroidal thickness was significantly higher in patients with KC (427.48 ± 78.51 μm) than in controls (351.03 ± 99.08 μm) [11]. Another paper showed a higher CMT in patients with KC (363.9 ± 59.8 μm and 328.4 ±  67.2 μm) [12]. However, no difference in CMT was reported in a study on 44 patients with KC and an equal number of healthy participants [20]. Similarly, we did not detect any significant changes between the CMTs in these two groups. Another study analyzed the CMT and electroretinographic findings in patients with KC. The CMT showed no difference. However, the retinal response density in multifocal electroretinography differed significantly [21]. Again, this may suggest impaired macular function.

Flatter corneal curvature was inversely associated with macular thickness in Asians [22]. However, Yang et al. found no correlation between corneal thickness and vascular densities in myopic patients [23].

KC staging had no impact on vascular density parameters (*p* = 0.17). Therefore, we can speculate that although we found a correlation between BCVA and OCT-A parameters, the analysis did not reveal any correlation between keratometry or CCT and vascular density.

Correlations between OCT-A parameters and the BCVA have been found in studies on other diseases, such as pathological myopia and diabetic retinopathy [24,25]. Furthermore, a correlation between retinal flow disorders and the integrity of the photoreceptors was demonstrated in a study performed by Scarinci et al. [26]. The importance of choroidal circulation in retinal function is supported by the fact that it supplies most of the outer retina, including the photoreceptors. Nevertheless, the contribution of the retinal circulation to photoreceptor metabolism is present [27]. As mentioned, the retinal and choroidal plexus influence visual acuity and retinal function; therefore, the demonstrated correlation between BCVA and superficial plexus density has justification and should be further investigated.

A possible mechanism for the vascular changes occurring in KC is basement membrane destruction. Mutations in TIMP-3 and several other genes have been observed in KC. TIMP-3 is an important gene involved in retinal pigment epithelium remodeling [2,28]. Lacquer cracks in the retina and subretinal hemorrhages with CNV were observed in a patient with bilateral KC with hydrops in one eye [29].

Similarly, lower vascular density was observed in non-glaucomatous eyes with exfoliation syndrome (XSF) than in healthy ones. Like KC, XFS is also a disorder characterized by basement membrane damage [30]. In a study on ocular sarcoidosis, Cerquaglia et al. observed lower vascular density in the retina and choriocapillaris in sarcoid eyes. Changes in the FAZ area were not significant [31]. The authors hypothesized that the differences mentioned above might be explained by inflammatory ischemia.

Traditional methods for detecting KC include slit-lamp examination and corneal topography using either a Scheimpflug camera or modern optical coherence tomography. However, since its introduction in diagnosing anterior segment diseases, OCT has become more and more helpful in diagnosing the KC [32,33]. Contrary to Scheimpflug technology, OCT offers high-resolution images that may help establish the diagnosis [33]. In addition, OCT angiography is a relatively new update to OCT that allows accurate imaging of the vasculature in the posterior pole without injecting a dye. This technology has proven to be helpful in studying both the physiology of the retina and disorders [34].

Various factors affect OCT-A density, such as signal strength [35], ethnicity [17], high myopia [36], and neurological diseases [16]. Contrary to choroidal thickness, circadian rhythm has little effect on the parafoveal density [37]. Furthermore, reports regarding age and gender effects on OCT-A parameters are contradictory [34,38,39,40].

A potential limitation of the current study is the inclusion of both eyes. However, it is possible to use a nested ANOVA model and have both eyes in the study [41,42]. Additionally, we only evaluated the vascular densities in the superficial plexus. However, Angioplex automatically provides information about only the superficial plexus. Secondly, in an animal study, Campbell et al. demonstrated that all the vascular layers merged into one to create a single FAZ [43]. Furthermore, we did not assess the posterior surface of the cornea. Although some studies have shown a correlation between axial length and vascular parameters, we did not measure axial length, as both of our groups were myopic [44,45].

In conclusion, our study suggests a significant decrease in macular vascular density in KC patients. Therefore, in the pathophysiology of visual impairment in KC, a macular vascular disorder that is not visible ophthalmoscopically may coexist with the corneal abnormality. Future studies should address the correlation between the severity of KC and vascular changes.

## Figures and Tables

**Table 1:** Qualitative variables of patients with keratoconus and healthy controls who qualified for the study. The data were compared using the chi-squared (c^2^) test. Statistical significance was set at *p* < 0.05.

Laterality		OS	OD	All Groups	c^2^	*p*
Group	Control	20	27	47	0.549	*p* = 0.459
Group	Keratoconus	39	40	79		
Row	Totals	59	67	126		
Gender		F	M	Totals		
Group	Control	16	12	28	3.127	*p* = 0.077
Group	Keratoconus	15	27	42		
Row	All Groups	31	39	70		

**Table 2:** Quantitative variables of patients with keratoconus and healthy controls who qualified for the study. Statistical significance was set at *p* < 0.05.

	Control Group		Keratoconus Group		
Variable	Mean	SD	Mean	SD	*p*
Age	42.40	16.99	43.22	14.24	*p* = 0.683
BCVA	0.94	0.12	0.63	0.30	*p* < 0.001
BCVA LogMAR	0.02	0.92	0.20	0.52	*p* < 0.001
K1	43.25	0.74	44.67	3.85	*p* = 0.002
K2	43.88	0.67	47.85	4.66	*p* < 0.001
FAZ area	0.25	0.09	0.20	0.12	*p* < 0.001
Perimeter	2.09	0.39	1.86	0.63	*p* = 0.001
Circularity	0.72	0.10	0.71	0.09	*p* =0.146
Central	8.44	3.33	6.78	4.74	*p* = 0.049
Inner	16.54	2.89	13.64	5.13	*p* = 0.002
Outer	16.88	2.42	14.71	4.12	*p* = 0.004
Full	16.57	2.48	14.25	4.30	*p* = 0.003
Epithelium center	48.83	7.34	44.52	7.02	*p* = 0.002
Epithelium min-max	−5.17	2.71	−10.22	6.22	*p* < 0.001
CCT	538.66	29.14	478.58	45.27	*p* < 0.001
CCTmin	518.26	36.78	425.49	106.86	*p* < 0.001
CMT	258.00	19.00	255.34	25.35	*p* = 0.936
Volume cube	10.01	0.62	11.33	10.23	*p* = 0.107
RNFL	90.26	7.74	91.16	14.28	*p* = 0.101
RNFL symmetry	81.56	11.83	72.78	22.16	*p* = 0.079
Rim area	1.29	0.20	1.49	0.36	*p* < 0.001
Disc area	1.71	0.36	2.02	0.39	*p* < 0.001
Cup volume	0.12	0.10	0.14	0.18	*p* = 0.603
c/d average	0.45	0.16	0.47	0.17	*p* = 0.232
Sphere	−1.75	2.02	−0.89	4.08	*p* = 0.163
Cylinder	−0.92	0.83	−3.30	2.46	*p * = 0.001
Axis	110.22	42.68	90.97	54.93	*p* = 0.400

**Table 3:** Pearson correlation matrix for tested variables in KC patients. Statistically significant values are marked in red.

	Group = Keratoconus																
Variable	BCVA	K2	Epi	CCT	CCT min	Faz Area	Perimeter	Circularity	Central	Inner	Outer	Full	CMT	RNFL	Cup Volume	Cylinder	Axis
BCVA	1	−0.64	0.19	0.3	0.17	−0.07	−0.15	0.15	0.41	0.44	0.37	0.4	0.09	0.15	0.18	0.38	0.05
K2	−0.64	1	−0.46	−0.34	−0.28	0.03	0.08	−0.13	−0.26	−0.3	−0.21	−0.24	−0.21	−0.06	−0.27	−0.54	−0.04
Epi	0.19	−0.46	1	0.51	0.35	0.01	0.05	−0.02	0.18	0.25	0.3	0.28	−0.15	0.32	0.07	0.52	−0.07
CCT	0.3	−0.34	0.51	1	0.77	0.08	−0.02	0.16	0.24	0.29	0.33	0.32	−0.11	0.07	0.05	0.43	−0.36
CCTmin	0.17	−0.28	0.35	0.77	1	0.19	0.09	0.08	0.11	0.25	0.3	0.28	−0.07	0.02	0.04	0.32	−0.35
Faz area	−0.07	0.03	0.01	0.08	0.19	1	0.93	−0.33	−0.29	0.05	0.16	0.12	−0.67	0.26	−0.29	−0.13	−0.21
Perimeter	−0.15	0.08	0.05	−0.02	0.09	0.93	1	−0.59	−0.34	0.01	0.11	0.07	−0.67	0.23	−0.3	−0.1	−0.17
Circularity	0.15	−0.13	−0.02	0.16	0.08	−0.33	−0.59	1	0.3	0.07	0.05	0.06	0.33	0.07	0.19	−0.02	0.03
Central	0.41	−0.26	0.18	0.24	0.11	−0.29	−0.34	0.3	1	0.87	0.81	0.84	0.47	0.21	−0.13	0.4	−0.14
Inner	0.44	−0.3	0.25	0.29	0.25	0.05	0.01	0.07	0.87	1	0.96	0.98	0.17	0.41	−0.25	0.48	−0.22
Outer	0.37	−0.21	0.3	0.33	0.3	0.16	0.11	0.05	0.81	0.96	1	1	0.05	0.49	−0.28	0.44	−0.31
Full	0.4	−0.24	0.28	0.32	0.28	0.12	0.07	0.06	0.84	0.98	1	1	0.1	0.46	−0.27	0.45	−0.29
CMT	0.09	−0.21	−0.15	−0.11	−0.07	−0.67	−0.67	0.33	0.47	0.17	0.05	0.1	1	−0.39	0.09	0.06	−0.02
volume cube	0.07	0.01	−0.15	0	0.04	−0.24	−0.24	0.08	0.05	−0.02	−0.06	−0.04	0.16	0.17	0.15	−0.06	0.15
RNFL	0.15	−0.06	0.32	0.07	0.02	0.26	0.23	0.07	0.21	0.41	0.49	0.46	0.39	1	0.03	0.19	0.04
Cup volume	0.18	−0.27	0.07	0.05	0.04	−0.29	−0.3	0.19	−0.13	−0.25	−0.28	−0.27	0.09	0.03	1	0.06	0.28
Cylinder	0.38	−0.54	0.52	0.43	0.32	−0.13	−0.1	−0.02	0.4	0.48	0.44	0.45	0.06	0.19	0.06	1	−0.12
Axis	0.05	−0.04	−0.07	−0.36	−0.35	−0.21	−0.17	0.03	−0.14	−0.22	−0.31	−0.29	−0.02	0.04	0.28	−0.12	1

Epi—epithelium center, CCT—central corneal thickness, CMT—central macular thickness, RNFL—retinal nerve fiber layer, BCVA—best-corrected visual acuity, FAZ—foveal avascular zone.

**Table 4:** Pearson correlation matrix for tested variables in healthy controls. Statistically significant values are marked in red.

	Group = Control									
Variable	CCTmin	Faz Area	Perimeter	Circularity	Central	Inner	Outer	Full	CMT	RNFL Symmetry
CCTmin	1	−0.68	−0.57	−0.56	0.87	0.03	0.19	0.19	0.72	0.28
Faz area	−0.68	1	0.99	0.87	−0.76	0.64	0.5	0.5	−0.97	0.44
Perimeter	−0.57	0.99	1	0.89	−0.71	0.69	0.55	0.56	−0.96	0.51
Circularity	−0.56	0.87	0.89	1	−0.58	0.61	0.47	0.49	−0.93	0.44
Central	0.87	−0.76	−0.71	−0.58	1	0	0.15	0.16	0.74	0.18
Inner	0.03	0.64	0.69	0.61	0	1	0.97	0.98	−0.6	0.92
Outer	0.19	0.5	0.55	0.47	0.15	0.97	1	1	−0.43	0.97
Total	0.19	0.5	0.56	0.49	0.16	0.98	1	1	−0.44	0.97
CMT	0.72	−0.97	−0.96	−0.93	0.74	−0.6	−0.43	−0.44	1	−0.35
volume cube	0.11	0.36	0.37	0.09	−0.22	0.37	0.47	0.43	−0.13	0.58
RNFL symmetry	0.28	0.44	0.51	0.44	0.18	0.92	0.97	0.97	−0.35	1

CCT—central corneal thickness, CMT—central macular thickness, RNFL—retinal nerve fiber layer, FAZ—foveal avascular zone.

## Data Availability

Data are available upon reasonable request.
